# Expected Value of Control and the Motivational Control of Habitual Action

**DOI:** 10.3389/fpsyg.2019.01812

**Published:** 2019-08-13

**Authors:** Andreas B. Eder, David Dignath

**Affiliations:** ^1^Department of Psychology, Julius Maximilian University of Würzburg, Würzburg, Germany; ^2^Department of Psychology, University of Freiburg, Freiburg, Germany

**Keywords:** habit, outcome devaluation, Pavlovian-to-instrumental transfer, default-interventionist framework, expected value of control, cognitive control

## Abstract

A hallmark of habitual actions is that, once they are established, they become insensitive to changes in the values of action outcomes. In this article, we review empirical research that examined effects of posttraining changes in outcome values in outcome-selective Pavlovian-to-instrumental transfer (PIT) tasks. This review suggests that cue-instigated action tendencies in these tasks are not affected by weak and/or incomplete revaluation procedures (e.g., selective satiety) and substantially disrupted by a strong and complete devaluation of reinforcers. In a second part, we discuss two alternative models of a motivational control of habitual action: a default-interventionist framework and expected value of control theory. It is argued that the default-interventionist framework cannot solve the problem of an infinite regress (i.e., what controls the controller?). In contrast, expected value of control can explain control of habitual actions with local computations and feedback loops without (implicit) references to control homunculi. It is argued that insensitivity to changes in action outcomes is not an intrinsic design feature of habits but, rather, a function of the cognitive system that controls habitual action tendencies.

“The chains of habit are too weak to be felt until they are too strong to be broken.” (adage credited to Samuel Johnson, 1748, “The vision of Theodore”)

Human beings like to view themselves as rationally behaving agents (Nisbett and Wilson, 1977). Yet, we are also creatures of habit. Accordingly, scientists in many different fields have been attracted to the study of habits because they invoke a dichotomy between automatic and controlled behavior (Wood and Rünger, 2016). A popular view is that habits run on autopilot until something goes wrong. For an illustration, let us take the example of our fictitious friend Tom: when he comes home from work, he has the habit to grab a can of cold beer from the fridge and to enjoy his after-work beer. On one unfortunate day, his wife bought the wrong beer, and the drama unfolds: Tom takes his usual large gulp, grimaces in distaste, and the moment is spoiled. What will happen to Tom? Will he continue with drinking, even if he cannot have his favorite beer? Maybe at a reduced rate? Or does he stop beer drinking all at once?

These questions are far from trivial, because behavior analysts commonly agree that habitual action is in principle and by definition independent of the current value of the produced outcome (see the next section). Yet, it is also clear that most people can control and correct habitual actions to some degree if the outcome is dysfunctional. In fact, a persistent inability to correct for unwanted habitual action patterns is a hallmark of a variety of pathological states (e.g., addiction)—and hence the atypical outcome of action control in healthy adults.

This article reviews research on the motivational control of habitual action. In a first section, we will discuss insensitivity to changes in action outcomes as a defining feature of habitual actions. Then, we will review behavioral and neuroscientific studies that examined a goal-independency of cue-instigated action tendencies with posttraining outcome revaluation procedures in operant learning and outcome-selective Pavlovian-to-instrumental transfer (PIT) tasks. In the second part, we will discuss two theoretical accounts: a default-interventionist framework and expected value of control (EVC) theory. While both accounts can explain a motivational control of habitual action, we will argue that EVC theory has more potential to provide a convincing account of habit control in PIT tasks.

## Part I

### Dual Action Psychology: Habitual and Goal-Directed Actions

According to behavior analysts, a *habit* is an acquired behavior that is triggered by an antecedent stimulus (Dickinson, 1985). Habit is distinguished from goal-directed action that is controlled by the current value of the action goal through knowledge about the instrumental relations between the action and its consequences. Often implicit to this distinction is an assignment of features of automaticity (e.g., associative, unintentional, efficient, etc.) to habitual actions and features of non-automaticity (e.g., rule-based, intentional, capacity-limited, etc.) to goal-directed actions (Dickinson and Balleine, 1993). However, close scrutiny of this distinction makes clear that this dichotomy is not justified and too simple (for thorough discussions, see Bargh, 1994; Moors and De Houwer, 2006; Keren and Schul, 2009; for counterarguments, see Evans and Stanovich, 2013). More useful seems a functional distinction based on correlations between actions and context features and correlations between actions and valued outcomes: instrumental actions are goal-directed because they are correlated more strongly with the presence or absence of desired outcomes than with the presence of particular contexts or stimuli. For example, if Tom drinks his after-work beer because he has a desire to get drunk, he would be willing to consume another alcoholic beverage if it has the same intoxicant effect. Habitual action, by contrast, is correlated more strongly with context features than with the presence or absence of a particular outcome of the action. For example, Tom would drink his after-work beer even if he is not thirsty or keen on getting drunk. For him, it is a behavioral routine that becomes activated in the appropriate context. That means, he would not have drunk the beer at another time or place, and assuming that he has developed a habit of beer drinking, even not another beverage.

At this point, a few additional qualifications are necessary. First, the correlation of habitual actions with particular contexts (or states) does not mean that they are unrelated to the value of these contexts. Habits typically arise from frequent repetitions of previously rewarded (instrumental) actions, that means, they often have a strong reward history (Yin and Knowlton, 2006). This rewarding context does not change with the performance of a habitual action (“Tom still gets drunk after beer consumption”) but, rather, the internal representation of this state as action outcome has changed (“getting drunk is a by-product and not an intended consequence anymore”). Complicating things further, a similar point can be made in respect to a correlation between instrumental actions and context features. Goal-directed actions are situated in particular contexts that offer a variety of informative cues for action control. Organisms exploit these cues in their active pursuit of a valued outcome and, if encountered on a regular basis, the action is correlated with the presence and absence of these contextual cues. Taken together, this means that a functional distinction between habitual and goal-directed actions based on the relative strength of correlations is gradual—and not a categorical one.

Second, for the analysis of a goal-dependency of actions, it is meaningful to distinguish between proximal and distal outcomes of actions. According to the standard definition, habitual action is not controlled by the value of proximal outcomes (“Tom does not drink his after-work beer because of the good taste of the beer”); however, the context in which the habit is performed is controlled by outcomes that are more distally related to the habitual behavior (e.g., “Tom wants to enjoy his leisure time and beer drinking serves this goal”). Thus, distal consequences can be causally involved in the performance of a habitual action even if its performance is insensitive to its immediate outcome. Note that this relationship implies a roughly hierarchical structure in which the habit (“beer drinking”) is nested in a more abstract and/or temporally extended activity (“enjoyment of the evening”). In the following, we mean an insensitivity to immediate outcomes when referring to a goal-independency of habits.

### Goal-Independency of Habits

Having laid out what habitual actions are, we now discuss studies examining a goal-independency of habitual actions. Given the extensive research literature on habit acquisition and performance, this review is necessarily selective. In the following, we will focus on laboratory studies with humans and animals in which reinforcing stimuli were devalued after extensive instrumental training. For example, devaluation treatments could be the pairing of a food reinforcer with toxin, or the devaluation of a monetary reinforcer. Critically, this devaluation was done after reinforcement learning; consequently, the value of the reinforcer was changed in the absence of the associated action. Following devaluation, action performance was tested in extinction (i.e., without presentation of the reinforcer that would have allowed for new reinforcement learning). If the animal or human continued to perform the behavior which had produced the now-devalued reinforcer, it was concluded that the motivation to perform this action was not driven by the current value of the reinforcer (i.e., action outcome)—and hence habitual.

First, it should be noted that many studies with posttraining devaluations of action outcomes found that actions do *not* become habitual even after extensive training (e.g., Adams and Dickinson, 1981). For example, a classic study trained rats to perform two distinct actions, each reinforced by a unique food reward (Colwill and Rescorla, 1985). After extensive training, one reward was devalued by pairing it with a toxin (flavor-aversion conditioning). Then, the animal was given the opportunity to engage in each of the responses in extinction. The study showed that the postlearning devaluation of the food reinforcer selectively reduced working for that food. Obviously, the rat had retrieved a memory of the devalued food outcome during the extinction test, in contradiction to early views that the reinforcer becomes not encoded in associative stimulus-response structures controlling reinforced behaviors (Thorndike, 1911; Hull, 1931). On the other hand, working for the devalued outcome was often not completely abolished in this research, which was viewed as evidence for habit formation. However, caution is warranted with this interpretation. First, other factors besides context features could have motivated the residual performance. For instance, the animal could have tested out whether the action will continue to produce no reinforcer in the extinction test (see research on the so-called “extinction burst”; Lerman and Iwata, 1995). Second, the devaluation of the reinforcer was most typically incomplete (Colwill and Rescorla, 1990). We will come back to this issue when we discuss the effectiveness of outcome devaluation treatments below.

Subsequent studies examined more specific conditions in which instrumental performance becomes insensitive to outcome values. This research suggested that overtraining, single-response training regimes, and interval-based reinforcement schedules (relative to a fixed-ratio schedule) are conducive to habit formation (e.g., Dickinson et al., 1983; Tricomi et al., 2009; Kosaki and Dickinson, 2010). However, even these protocols do not invariably lead to an insensitivity outcome values (for a recent failure, see de Wit et al., 2018) and the conditions necessary for habit formation are still not very well understood (Hogarth, 2018). Most important, the ideal “habit test” examines not only an insensitivity to correlations with (de)valued outcomes but also a sensitivity to correlations with context features. This test is found in a procedure called *outcome-selective Pavlovian-to-instrumental transfer of control* (PIT).

**Figure 1:**
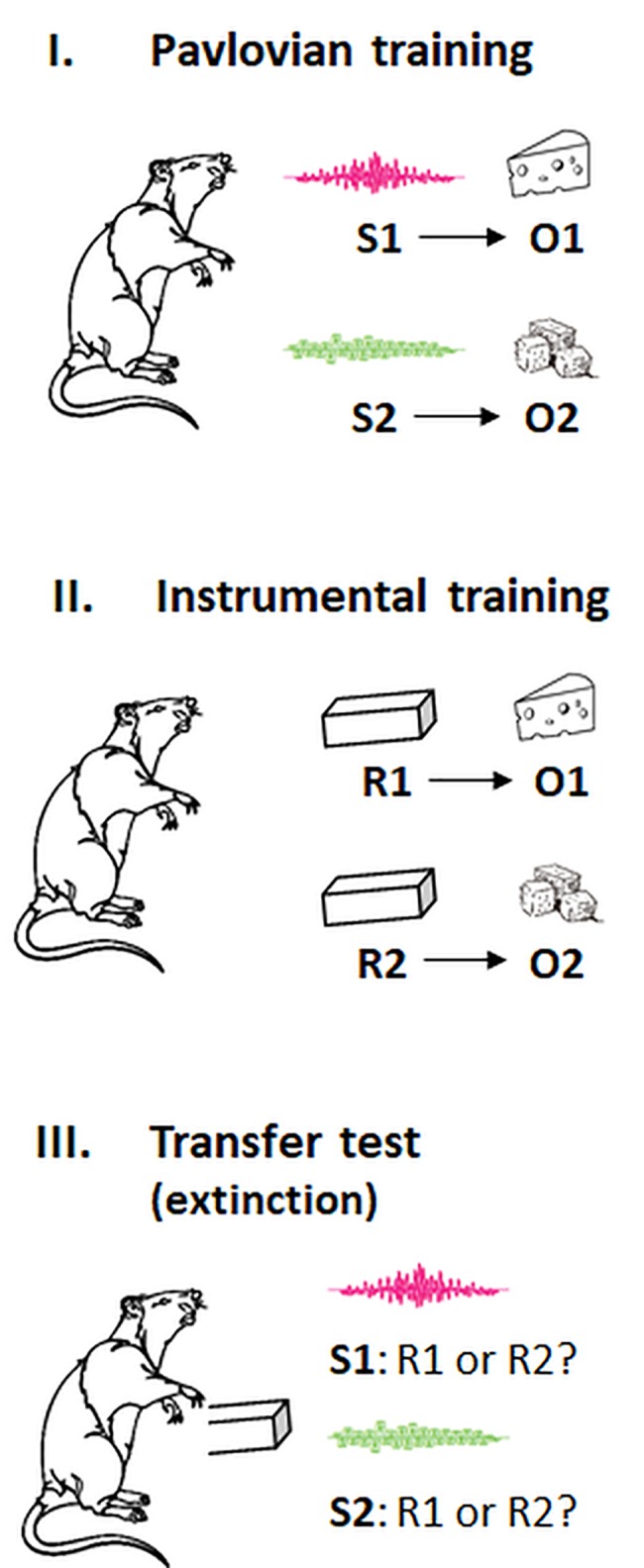
Pavlovian, instrumental, and transfer phases in the outcome-selective PIT paradigm. S, stimulus cue; R, response; O, outcome. The animal shows no preference for a particular outcome (here: two flavors of cheese) before the training. In the transfer test, the response associated with the same outcome as the stimulus cue is typically preferred (i.e., S1: R1 > R2; S2: R2 > R1). See the text for more explanation.

In outcome-selective PIT, stimuli that are predictive of specific outcomes prime instrumental responses that are associated with these outcomes. The canonical procedure is shown in Figure 1 and consists of three separate phases: an a first *Pavlovian training phase*, participants learn predictive relations between stimuli and differential outcomes (e.g., S1-O1, S2-O2). In a subsequent *instrumental training phase*, they learn to produce these outcomes with particular actions (e.g., R1-O1, R2-O2). In a *transfer test*, both actions are then made available in extinction, and the preference for a specific action is measured in the presence of each conditioned stimulus (i.e., S1: R1 or R2?; S2: R1 or R2?). The typical result is a preference for the action whose outcome was signaled by the Pavlovian cue (i.e., S1: R1 > R2; S2: R2 > R1), suggesting that this stimulus has gained control over responding (for a review and meta-analysis, see Holmes et al., 2010; Cartoni et al., 2016). Note that this cue-instigated action tendency cannot be explained with rote S-R learning because the action was not paired with the Pavlovian cue before the transfer test. Instead, it has been suggested that the Pavlovian cue primes the action by activating the sensory representation of the associated outcome *via* an associative S:(R-O) or S:(O-R) chain (Trapold and Overmier, 1972; Asratyan, 1974; Balleine and Ostlund, 2007; de Wit and Dickinson, 2009). According to this account, the Pavlovian cue activates a cognitive representation of the identity of the outcome (whatever its value), and this activation excites the action that is associated with the same outcome. In line with an associative S-O-R mechanism, research on “ideomotor effects” showed that presentations of action effect-related stimuli prime actions producing these effects (for reviews, see Shin et al., 2010; Hommel, 2013). An alternative account proposed that the Pavlovian cues act like discriminative stimuli in a hierarchical network that signal when a specific R-O relationship is in effect (Cartoni et al., 2013; Hogarth et al., 2014). According to this account, action choice in PIT tasks is driven by participants’ explicit beliefs about which action is more likely reinforced in the presence of a specific cue. For instance, participants in one experiment were told that the cues presented during a PIT test would indicate which action would *not* be rewarded. This instruction reversed the cue-instigated action tendency (Seabrooke et al., 2016). A follow-up study found this reversed PIT effect abolished by a cognitive load manipulation, while the standard PIT effect was spared (Seabrooke et al., 2019b). This research suggests that several processes could contribute to outcome-selective PIT effects: a resource-dependent one that is highly amenable to instructions, and a relatively resource-independent one that could be an association-based mechanism or a very simple behavioral rule. It should be noted that outcome-selective PIT effects were also observed in rodent studies, and it has been argued that the underlying mechanisms are causally involved in a broad range of “habitual” behaviors (Everitt and Robbins, 2005; Watson et al., 2012; Hogarth et al., 2013; Colagiuri and Lovibond, 2015).

Importantly, the outcome-selective PIT task can be combined with outcome devaluation treatments to examine a goal-independency of cue-instigated action tendencies. Using this research approach, animal studies found that rodents still work harder for a devalued food in the presence of a Pavlovian or discriminative cue associated with that food (Rescorla, 1994; Corbit and Balleine, 2005; Corbit et al., 2007). For example, in one study (Holland, 2004), hungry rats learned relations between stimuli and two unique food rewards (sucrose and food pellets). These food rewards were then used to reinforce two distinct actions (chain pulling and lever presses). In a subsequent extinction test, the rats had access to these responses during presentations of the Pavlovian cues. Performance in this first transfer test showed a standard outcome-selective PIT effect. After this test, one of the two food rewards was devalued by pairing it with a toxin. Then, the rats worked on a second transfer test in extinction. Although the conditioned food aversion clearly decreased working for that food at baseline, the cue-instigated action tendency augmenting the devalued response was spared.

Results of outcome-selective PIT studies with human adults were however more mixed. While some studies confirmed the finding of animal studies that reinforcer-selective PIT does not change when the outcome is no longer desirable (Hogarth and Chase, 2011; Hogarth, 2012; Watson et al., 2014; van Steenbergen et al., 2017; De Tommaso et al., 2018), a few studies observed a change. One of these studies used a stock-market paradigm for a postlearning devaluation of outcomes (Allman et al., 2010). Human adults first learned to associate specific symbols and instrumental actions with two (fictitious) money currencies. In this phase of the experiment, both currencies had the same value, and participants knew that they can swap the earnings into real money after the study. In a first extinction test, a clear PIT effect was observed. After retraining, and immediately before a second transfer test, one of the two currencies was devalued by making the currency worthless. In the subsequent extinction test, responding for the intact currency was still elevated by matching cues; in contrast, working for the devalued money was generally disrupted and not affected by presentations of a matching cue. In short, the Pavlovian cue had lost its capacity to excite the devalued action.

Follow-up research showed that the cue-instigated action tendency is affected by a postlearning value decrease, but not by an equidistant value increase (Eder and Dignath, 2016a). The study used a stock-market paradigm similar to Allman et al. (2010). This time, however, the revaluation treatment involved three monetary outcomes: one currency was made worthless as in Allman et al. by decreasing its value by one unit (1 → 0); the value of another currency was doubled (1 → 2); the third currency maintained its value (1 → 1) for baseline comparisons. If the cue-instigated action tendency is truly sensitive to the current value of outcomes, then it should decrease following the devaluation but increase following the upvaluation of the outcome. Results however showed that only the devaluation treatment had an effect: Outcome-selective PIT was significantly reduced after devaluation, reproducing the result of Allman et al. (2010). In contrast, PIT effects were not different from the baseline condition after the upvaluation. In short, only a decrease in the outcome value affected cue-instigated action tendencies, while an equidistant value increase had no effect.

The PIT studies reviewed above are puzzling and at odds with a large number of studies that reported no effect of postlearning changes in outcome values. In the search for an explanation, Watson and colleagues proposed that the stock-market paradigm involved highly abstract representations of values that were presumably more accessible to explicit choice strategies (Watson et al., 2018). While it is unclear why those explicit decision rules should not take a value increment into account (see Eder and Dignath, 2016a), recent studies confirmed that explicit beliefs can have a profound impact on outcome-selective PIT effects (see e.g., Seabrooke et al., 2016). In addition, the theoretical argument was made that Pavlovian cues can only activate the sensory identity of action outcomes in PIT tasks and not their value (Balleine and Ostlund, 2007; de Wit and Dickinson, 2009). If money outcomes in the stock-market studies were represented predominantly in terms of their value, this could have made a critical difference to (animal) studies that used primary reinforcers with a more detailed sensory representation. Accordingly, it could be hypothesized that a standard PIT task with food outcomes should be not sensitive to postlearning changes in the values of outcomes.

**Figure 2:**
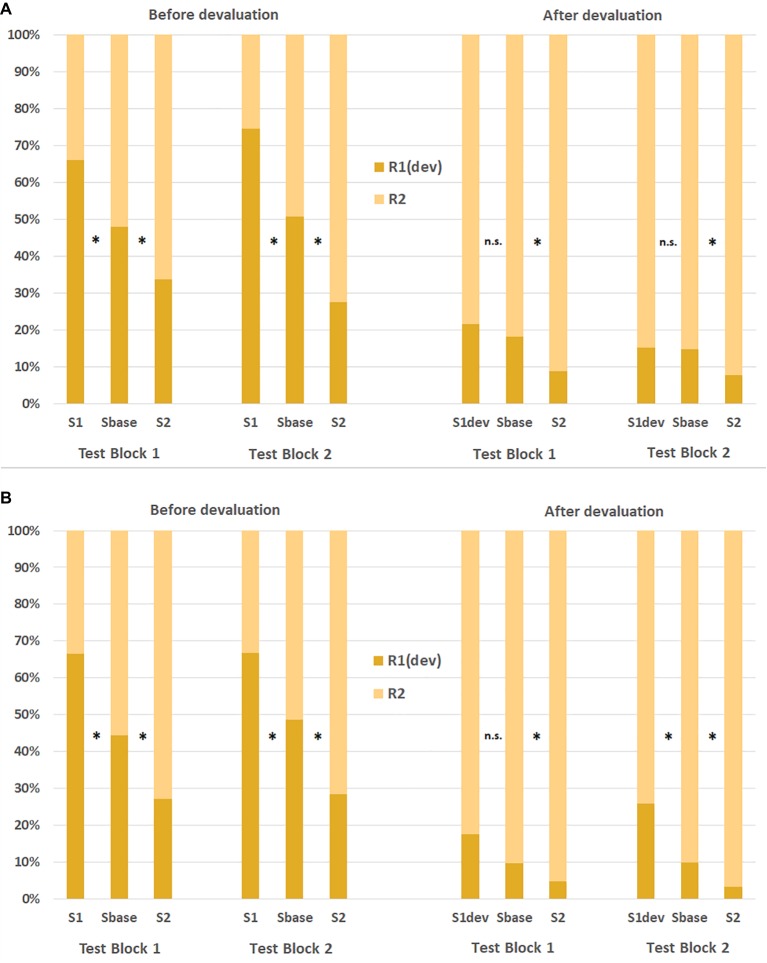
Response ratios in Eder and Dignath (2016b) before and after the devaluation of a liquid reinforcer as a function of stimulus cue, action, and test block in Experiment 1 (upper panel A) and Experiment 2 (lower panel B). S, stimulus cue; R, response; dev, devalued outcome. ^*^significant difference to the baseline condition at *p* < 0.05.

Eder and Dignath (2016b) tested this hypothesis with liquid reinforcers. Participants were trained in separate sessions to associate specific symbols and keypresses, respectively, with red and yellow lemonades. Importantly, participants in this study were asked to consume the lemonades earned during a transfer test^1^. After having worked on a first transfer test, one of the lemonades was devalued with bad-tasting Tween20. Then, a second transfer test was performed. Each transfer test was further subdivided into two test blocks. In the first experiment, participants consumed the earned lemonades immediately after each test block. In the second experiment, consumption was not immediate, and participants could take the earned lemonades with them in bottles. Figure 2 shows the response rates in both experiments as a function of the Pavlovian cue in each test block. As can be seen, a strong and robust PIT effect was observed in both experiments before devaluation: working for a specific lemonade was elevated by presentations of cues associated with that lemonade (relative to a baseline condition with a neutral cue associated with no lemonade). However, response rates changed dramatically following the devaluation. Participants now preferred the action that produced the intact lemonade. Responding for this lemonade was still augmented by a matching cue relative to baseline. In contrast, the cue-instigated action tendency was abolished for the devalued response in Experiment 1 in which the liquids earned in a test block were consumed immediately. Interestingly, in Experiment 2 (without immediate consumption of the liquids), the cue-instigated tendency for the devalued response was abolished in the first test block only and restored in the second test block^2^. It is plausible that the immediate consumption of the drinks increased the motivational relevance of the devalued drink. These results hence show that a strong devaluation treatment of food outcomes can also reduce cue-instigated action tendencies operating on a primary reinforcer.

For an explanation, Eder and Dignath (2016b) suggested that only strong devaluation treatments suppress cue-instigated instigated actions. In fact, most studies that found no effect of the devaluation treatment used rather weak and/or incomplete devaluation treatments, such as ad libitum feeding, conditioning of a taste aversion, or health warnings (for a similar argumentation, see De Houwer et al., 2018)^3^. Hogarth and Chase (2011), for instance, used a specific satiety procedure to devalue a tobacco outcome. Although smoking a cigarette before a transfer test reduced participants’ craving and working for cigarettes during the PIT test, cue-instigated action tendencies for that reward were not affected. Critically, working for the devalued tobacco outcome (irrespective of the cue) was still on a high level (>40%), suggesting that the devaluation was not very strong. In addition, regular smokers typically know that the state of satiety is only temporary. Therefore, it could be argued that working for cigarettes was still attractive for them during the transfer test. The devaluation treatment that is most comparable to the one used by Eder and Dignath (2016b) is conditioning of a taste aversion. Rodent studies often devalued a food reinforcer by pairing it with lithium chloride (LiCl) inducing sickness (e.g., Rescorla, 1994; Holland, 2004). Although LiCl-conditioning has a strong and lasting effect on the consumption of that food, the devaluation is often incomplete, because the animal must approach a magazine to consume the poisoned food and could reject consumption before the devaluation was complete. In fact, when Colwill and Rescorla (1990) used a standard procedure to devalue a sucrose solution with LiCl-injections before a transfer test, the devaluation treatment did not eliminate the cue-instigated action tendency. However, when the poisoned sucrose solution was injected directly into the mouth of the rat during conditioning, the stimulus lost its capacity to elevate the devalued response. Thus, animal research also found cue-instigated action tendencies abolished after a strong and immediate devaluation treatment, in line with the results of human studies reviewed above.

Our main conclusion from this short review is that the cue-instigated action tendency was suppressed when the devaluation of the associated action outcome was strong and complete. This does not mean that the action tendency scales directly with the current value of the associate outcome, as proposed for a goal-directed process. In this case, studies with a weak (but still effective) devaluation of the outcome should also have observed a reduction in cue-instigated tendencies, which was not the case (e.g., Hogarth and Chase, 2011; Watson et al., 2014; De Tommaso et al., 2018). In addition, an upvaluation of the associated outcome should have enhanced the cue-instigated action tendency, which was not observed (Eder and Dignath, 2016a). In short, the studies reviewed above do not question that the cue-instigated action tendency was “habitual” in the sense that the behavior was insensitive to the current value of the outcome; rather, they suggest that the habitual action tendency was cognitively suppressed because the devalued outcome was in conflict with other goals or intentions. According to this interpretation, an internal conflict signal is created after registration that the present state will deteriorate markedly with continued performance of the habitual action. Detection of this conflict signal then triggers behavioral adaptations that aim to correct for the maladaptive habitual response. In the next section, we will describe two frameworks of how such a control system could be implemented on the cognitive level: a default-interventionist framework and EVC theory.

## Part II

In this part, we will discuss two alternative frameworks of cognitive control: (1) a *default-interventionist framework* that proposes a higher order cognitive control system that intervenes when the habitual action goes faulty. (2) *EVC theory* that explains the allocation of control with neural computations of the expected payoffs from engaging in cognitive control.

### Default-Interventionist Framework

The default-interventionist framework postulates a cognitive control system that can intervene when the habitual “default” response becomes inappropriate, cumbersome, or defective. In its most basic form, the framework assumes two systems or control units of actions: a habitual controller and a goal-directed controller. Only the goal-directed controller is sensitive to changes in outcomes, while the habitual controller implements a stimulus-driven behavior without detailed representation of its consequences. This distinction is supported by neurophysiological research that studied dissociations in the control of voluntary and habitual actions on a neural systems level. More specifically, habitual and goal-directed controllers have been linked to two distinct (but interacting) cortico-basal ganglia networks in the brain: The associative cortico-basal ganglia loop controls goal-directed actions *via* projections from the prefrontal cortex (PFC) to the caudate nucleus and the anterior putamen. The sensorimotor loop controls habitual actions and connects the somatosensory and motor cortex with the medial and posterior putamen (for reviews, see Yin and Knowlton, 2006; Balleine et al., 2007; Graybiel and Grafton, 2015). Research found that after overtraining of a response (i.e., habit formation), neural activation is shifted from the associative loop to the sensorimotor loop (Ashby et al., 2010). Interestingly, goal-oriented behavior can be reinstated after inactivation of the infralimibic prefrontal cortex in the rodent brain (Coutureau and Killcross, 2003). This finding suggests that the circuits controlling goal-directed behavior are actively suppressed after habit formation.

The default-interventionist framework rests on the idea that there is a dynamic balance between action control systems, and that control could be shifted back from the habitual to the goal-directed control system if needed. This idea also fits with the long-standing view that prefrontal cortical areas have the capacity to override unwanted lower-order action tendencies (Koechlin et al., 2003). However, it has been argued that regaining control over habitual action tendencies is effortful and requires cognitive resources (Baddeley, 1996; Muraven and Baumeister, 2000). Furthermore, the person must be sufficiently motivated to invest resources in the executive control of the habitual action (Inzlicht and Schmeichel, 2012). Hence, a number of requirements must be met for the default-interventionist framework (for a defense and criticisms of this view, see Evans and Stanovich, 2013; Kruglanski, 2013; Hommel and Wiers, 2017; Melnikoff and Bargh, 2018).

It is likely that these conditions were met in the posttraining devaluation studies reviewed above. With a strong and complete devaluation of the outcome, participants were arguably motivated to avoid that outcome. In addition, performing the free-operant transfer task was very easy and without time pressure. However, the explanatory problems with the default-interventionist framework are much more fundamental and concern the very architecture of this account. Specifically, it is not specified what controls the controller, leading to an infinite logical regress. This problem became apparent in early accounts that conceptualized the interventionist as a unitary system (supervisory attentional system, working memory system, goal-directed action controller, etc.,). This approach was heavily criticized of introducing a “homunculus” (the executive controller) that pulls the levers to regulate lower levels if needed (Monsell and Driver, 2000). As a reaction to this criticism, the unitary control system view was replaced by more complex models that decomposed the “executive” in more specific control functions (e.g., mental set shifting, memory updating, response suppression; Miyake et al., 2000). However, as Verbruggen et al. (2014) unerringly pointed out, this approach only resulted in a multiplication of control homunculi and not in an explanation of how control is exercised. Thus, a fundamentally different approach is needed that explains cognitive control functions as an emergent phenomenon of the cognitive system.

### Expected Value of Control

**Figure 3:**
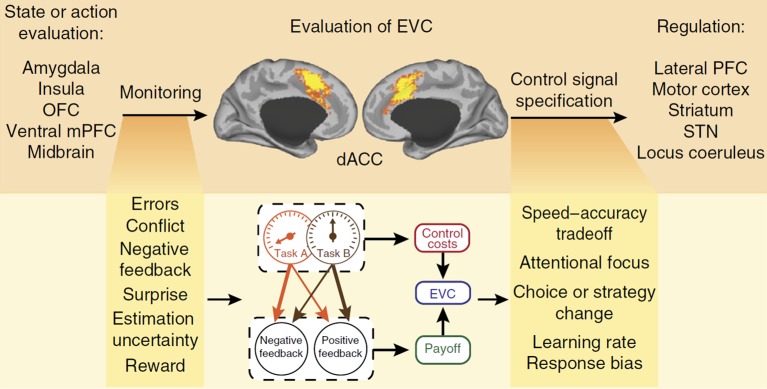
Control allocation according to EVC theory. The dACC monitors ongoing processes for signals relevant to evaluating EVC and specifies the optimal control allocation to downstream regions for overriding a default behavior. OFC, orbitofrontal cortex; STN, subthalamic nucleus; mPFC, medial prefrontal cortex; PFC, prefrontal cortex. Figure reprinted by permission from Springer Nature: *Nature Neuroscience*, “Dorsal anterior cingulate cortex and the value of control”, © Shenhav et al. (2016).

A model that has the potential to explain habit control in the PIT paradigm without recourse to control homunculi is found in EVC theory (Shenhav et al., 2013, 2016). This model analyzes cognitive control as a domain of reward-based decision making; that means, it is assumed that cognitive control functions serve to maximize desired outcomes through “controlled” processes when those outcomes could not otherwise be achieved by (habitual) “default” processes (Botvinick and Braver, 2015). The model aims to explain whether, where, and how much cognitive control is allocated to ongoing or planned activities. At the neural level, it is assumed that a central hub in this decision making process is the dorsal anterior cingulate cortex (dACC) that lies on the medial surfaces of the brain’s frontal lobes (see the central panel in Figure 3). Many studies showed that the dACC becomes active in control-demanding situations in which automatic action tendencies, such as habits, are in conflict with task-defined responses (see e.g., Procyk et al., 2000; for meta-analyses see Ridderinkhof et al., 2004; Nee et al., 2007). As a key hub in a wide network of distributed brain regions, it receives inputs from brain areas responsible for the valuation of incoming stimuli or action outcomes and sends output signals to areas responsible for the implementation of control (see Figure 3). In this network, it is assumed that dACC serves several functions: (1) it monitors ongoing processing to signal the need for control; (2) it evaluates the demands for control; (3) and it allocates control to downstream regions (Botvinick, 2007; Shenhav et al., 2016); for a different account of dACC functions, see Kolling et al., 2016).

According to EVC theory, two sources of value-related information are integrated in the dACC: (1) what control signal should be selected (i.e., its identity) and (2) how vigorously this control signal should be engaged (i.e., its intensity). The integration process considers the overall payoff that can be expected from engaging in a given control signal, taking into account the probabilities of positive and negative consequences that could result from performing a task. In addition, it takes into account that there is an intrinsic cost to engaging in control itself, which is a monotonic function of the intensity of the control signal (Shenhav et al., 2017). The expected value of a candidate control signal is the sum of its anticipated payoffs (weighted by their respective probabilities) minus the inherent cost of the signal (a function of its intensity). By relative comparisons, the candidate control signal with the maximum expected value is selected for a down-stream regulation of more basic processes. This selection process has been simulated as a stochastic evidence accumulation process using the drift diffusion model that avoids any recourse to a homunculus (Musslick et al., 2015). In contrast to the default-interventionist framework, EVC theory does not assume a hierarchy of action control systems but, rather, views the control of habitual actions as an emergent phenomenon of a unitary cognitive system. In addition, neural computations of the expected payoffs are continuously performed during task engagement, and control (e.g., attention) can be applied in varying degrees to the task at hand. It should be noted that the hypothesis of a neural implementation in the dACC is in principle independent of the computations proposed by the theory on the algorithmic level (Marr, 1982). In other words, it is possible that future neuroscientific research will identify other neural structures that calculate expected payoffs of engaging in control. By providing a computationally coherent and mechanistically explicit account of cognitive control functions on the algorithmic and implementational levels, EVC theory avoids the pitfall of introducing a new homunculus-like entity that magically guides cognition and behavior.

EVC theory can account for cognitive control functions and subsequent control adaptations in classic response conflict tasks (Ridderinkhof et al., 2004; Carter and van Veen, 2007; Nee et al., 2007), and the model was also used to explain behavioral flexibility that is characteristic of exploration and foraging (Shenhav et al., 2016). Most important for the present discussion, EVC theory can help to understand habit control in PIT tasks. In the remainder of this article, we provide a preliminary account of control functions in outcome-selective PIT.

In PIT tasks, the default response that must be potentially overcome is the cue-instigated action tendency that primes actions associated with shared outcomes. Before the revaluation treatment, however, there exists no motivation to override this default tendency. There is no action that would be more “correct” or valuable and that could be increased for a better payoff. To the contrary, overcoming the PIT tendency would be effortful (for indirect evidence on this assumption, see Cavanagh et al., 2013; Freeman et al., 2014; see also Yee and Braver, 2018). Therefore, the expected payoff does not justify the intrinsic cost of control. As a result, the cue-instigated action tendency is not or only minimally controlled in this phase, resulting in a PIT effect.

Expected payoffs however change dramatically after a strong revaluation of the outcome. Now, there exists a clear difference in the value of action outcomes, and response rates are adjusted to maximize the reward. At the computational level, this behavioral adjustment is implemented by prioritizing control signals that maximize the value of outcomes. As a consequence, control of action tendencies that would produce devalued outcomes is now justified, because the anticipated outcome of the intact response outweighs the effort that is necessary to override the devaluated response. Control is however not intensified following the registration of an action tendency that would result in high-value outcomes. As a consequence, the cue-instigated action tendency is only controlled (i.e., suppressed) if it results in a devalued outcome, whereas actions resulting in desirable outcomes do not (or to a much smaller degree) demand control.

EVC theory can hence explain why studies found reduced PIT tendencies only with very strong and/or complete devaluation treatments. The outcome value arguably shrank less by a weak relative to a strong devaluation treatment. The small decrement in the expected payoff does not justify the intrinsic costs of engaging in control. Furthermore, a EVC account of the PIT task can also explain observed effects that the default interventionistic account cannot explain. For instance, computations of expected payoffs take into account a temporal discounting of future and/or past outcomes (Yi et al., 2009). Immediate outcomes are typically weighted more than temporally distant outcomes. This immediacy bias can explain why immediate (relative to delayed) consumptions had a stronger effect on cue-instigated action tendencies in the study of Eder and Dignath (2016b). Furthermore, if the negative value of the devalued drink was discounted with the time that elapsed or will elapse since the consumption of that drink (Yi et al., 2009), the expected value of engaging in control is the largest immediately after consumption of the drink. Temporal discounting of the negative outcome value can hence explain why PIT tendencies were abolished in the first test block and restored in the second test block of Eder and Dignath’s experiment.

EVC theory also provides an explanation why the postlearning devaluation of the outcome had a stronger effect on the control of PIT tendencies compared to the upvaluation (Eder and Dignath, 2016a). Research on cognitive control showed that negative outcomes elicit a stronger control signal (Hajcak et al., 2005) and that conflict is aversive (Botvinick, 2007; Inzlicht et al., 2015). In line with this suggestion, studies found that conflict elicits a negative affective response (Dreisbach and Fischer, 2012) that triggers avoidance (Dignath et al., 2015; Dignath and Eder, 2015). In addition, (unexpected) positive events reduce conflict-driven behavioral adaptations, presumably because they weaken the negative conflict signal that signals need for control (e.g., van Steenbergen et al., 2009; but see also Dignath et al., 2017). It is hence plausible that a positive affective response to the (unexpected) upvaluation of a currency in the study of Eder and Dignath (2016a) has analogously decreased the intensity of the control signal that signaled need for control of the cue-instigated action tendency.

In summary, EVC theory can explain most findings of the PIT studies reviewed above. While this account is ex post facto, it has the benefit of providing a formal and mechanistic account of the effect of posttraining revaluation treatments on PIT tendencies. In addition, the account allows for new predictions. According to EVC theory, cognitive control of cue-instigated action tendencies should be inversely related to the intrinsic cost of control effort. Therefore, one would expect that PIT tendencies should recover in demanding transfer tasks with high intrinsic costs of control, even when the devaluation of the associated outcome was very strong. For instance, costs of engaging in control could be manipulated by increasing the investment of resources that are necessary to reach a decision and/or to implement the action (Boureau et al., 2015). These costs could be cognitive (e.g., evaluation times), physical (e.g., energy expenditure), and/or emotional (e.g., negative affective experiences). When intrinsic costs outweigh the cost of producing a devalued outcome in a PIT task, the prediction would be that control of cue-instigated action tendencies becomes relaxed, resulting in larger outcome-selective PIT effects. Having a strong foundation in neuroscientific research, the account also makes new predictions at the neural level. Specifically, activity of dACC should increase following the strong devaluation of an outcome, indexing the monitoring and implementation of a control setting. In addition, dACC should be most active during presentations of Pavlovian cues predictive of the devalued outcome. Hence, several hypotheses can be deduced from EVC theory that could be examined in future research.

## Conclusion

Habits have a great influence on our behavior. Some habits we strive for, and work hard to make them part of our behavioral repertoire. Other habits we want to abolish because they are problematic. Habits are consequently closely linked to cognitive control functions that regulate habitual action tendencies for the pursuit of higher-order goals. In this article, we argued on the basis of EVC theory that the allocation of control to habitual action tendencies is based on evaluations that compute the expected value of control by taking intrinsic costs of effortful control into account. Habits hence may be insensitive to changes in outcomes values because the expected benefits that follow from habit control do not justify the costs of control. The often cited insensitivity to changes in action outcomes is consequently not an intrinsic design feature of habits but, rather, a function of the cognitive system that controls habitual action tendencies.

## Author Contributions

AE drafted the manuscript. DD provided critical revisions. All authors approved the final version of the manuscript for submission.

### Conflict of Interest Statement

The authors declare that the research was conducted in the absence of any commercial or financial relationships that could be construed as a potential conflict of interest.

## Footnotes

^1^The transfer test was carried out in nominal extinction (i.e., without feedback whether or which lemonade had been earned). This was done to prevent the feedback from influencing the response choice. Instruction explicitly stated that the actions during the transfer test procure lemonades (2.5 ml according to the fixed-ratio 9 schedule) and that the probability of a reward is not influenced by the pictures presented during this phase. Note that a reward expectancy during the extinction test is common in PIT studies (see e.g., Hogarth and Chase, 2011; Colagiuri and Lovibond, 2015). Furthermore, it increases the ecological validity of the PIT task to behavior outside of the laboratory (for a discussion of this point see Lovibond and Colagiuri, 2013).

^2^Collapsed across both test blocks, however, there was small PIT effect for the devalued response. Furthermore, the magnitudes of the PIT effects for the devalued response in both test blocks were not significantly different.

^3^A notable exception is Experiment 1 in Seabrooke et al. (2017) that showed a PIT effect despite the use of a fairly strong devaluation treatment (coating of snacks with a distasteful paste). It should be noted, however, that (1) this study presented pictures of the food outcomes (and not Pavlovian cues) during the transfer test; (2) despite a clear reduction in subjective liking ratings, working for the devalued food (in the baseline condition) was still on a sizeable level (~25%); (3) the devalued food earned during the test was not immediately consumed (see Eder and Dignath, 2016b); (4) the same devaluation treatment affected PIT tendencies in subsequent experiments after modification of the task procedure (Seabrooke et al., 2017, 2019a).
